# CD151 supports VCAM-1-mediated lymphocyte adhesion to liver endothelium and is upregulated in chronic liver disease and hepatocellular carcinoma

**DOI:** 10.1152/ajpgi.00411.2016

**Published:** 2017-05-04

**Authors:** James C. R. Wadkin, Daniel A. Patten, Sivesh K. Kamarajah, Emma L. Shepherd, Vera Novitskaya, Fedor Berditchevski, David H. Adams, Chris J. Weston, Shishir Shetty

**Affiliations:** ^1^Birmingham Liver Biomedical Research Unit Institute of Immunology and Immunotherapy, National Institute for Health Research, University of Birmingham, Birmingham, United Kingdom;; ^2^CRUK Institute for Cancer Studies, Institute of Cancer and Genomic Sciences, University of Birmingham, Birmingham, United Kingdom; and; ^3^Liver Unit, University Hospitals Birmingham NHS Foundation Trust, Queen Elizabeth Hospital, Birmingham, United Kingdom

**Keywords:** tetraspanin 24, hepatic sinusoidal endothelial cells, lymphocyte recruitment

## Abstract

CD151, a member of the tetraspanin family of receptors, is a lateral organizer and modulator of activity of several families of transmembrane proteins. It has been implicated in the development and progression of several cancers, but its role in chronic inflammatory disease is less well understood. Here we show that CD151 is upregulated by distinct microenvironmental signals in a range of chronic inflammatory liver diseases and in primary liver cancer, in which it supports lymphocyte recruitment. CD151 was highly expressed in endothelial cells of the hepatic sinusoids and neovessels developing in fibrotic septa and tumor margins. Primary cultures of human hepatic sinusoidal endothelial cells (HSECs) expressed CD151 at the cell membrane and in intracellular vesicles. CD151 was upregulated by VEGF and HepG2 conditioned media but not by proinflammatory cytokines. Confocal microscopy confirmed that CD151 colocalized with the endothelial adhesion molecule/immunoglobulin superfamily member, VCAM-1. Functional flow-based adhesion assays with primary human lymphocytes and HSECs demonstrated a 40% reduction of lymphocyte adhesion with CD151 blockade. Inhibition of lymphocyte adhesion was similar between VCAM-1 blockade and a combination of CD151/VCAM-1 blockade, suggesting a collaborative role between the two receptors. These studies demonstrate that CD151 is upregulated within the liver during chronic inflammation, where it supports lymphocyte recruitment via liver endothelium. We propose that CD151 regulates the activity of VCAM-1 during lymphocyte recruitment to the human liver and could be a novel anti-inflammatory target in chronic liver disease and hepatocellular cancer prevention.

**NEW & NOTEWORTHY** Chronic hepatitis is characterized by lymphocyte accumulation in liver tissue, which drives fibrosis and carcinogenesis. Here, we demonstrate for the first time that the tetraspanin CD151 supports lymphocyte adhesion to liver endothelium. We show that CD151 is upregulated in chronic liver disease and hepatocellular carcinoma (HCC) and is regulated on endothelium by tissue remodeling and procarcinogenic factors. These regulatory and functional studies identify CD151 as a potential therapeutic target to treat liver fibrosis and HCC.

## Abstract (precis)

Chronic hepatitis is characterized by lymphocyte accumulation in liver tissue, which drives fibrosis and carcinogenesis. Here, we demonstrate for the first time that the tetraspanin CD151 supports lymphocyte adhesion to liver endothelium. We show that CD151 is upregulated in chronic liver disease and hepatocellular cancer (HCC) and is regulated on endothelium by tissue remodeling and procarcinogenic factors. These regulatory and functional studies identify CD151 as a potential therapeutic target to treat liver fibrosis and HCC.

chronic inflammatory liver disease can progress to end-stage liver failure and also promotes a protumorigenic environment, leading to hepatocellular carcinoma (HCC) (6, 10, 13, 26). Liver disease is a major global health burden, and HCC is now the second leading cause of cancer-related deaths worldwide (19). Inflammatory liver diseases are characterized by a chronic lymphocytic infiltrate, which drives the development of fibrosis and cirrhosis (21, 31). In contrast to other organs, in which extravasation usually occurs in postcapillary venules, the majority of lymphocyte recruitment to the liver occurs within the hepatic sinusoids, which are lined by highly specialized hepatic sinusoidal endothelial cells (HSECs) (9, 37). Control of the inflammatory response by HSECs through regulation of recruitment of recirculating lymphocytes is therefore central to tissue homeostasis, and any perturbation of endothelial cell function may drive the liver phenotype toward cirrhosis or tumorigenesis (6, 26). The low-shear hepatic sinusoids provide a unique environment for lymphocyte recruitment, in which HSECs display distinct differences in adhesion molecule expression, such as a lack of expression of P- and E-selectin, compared with conventional vascular endothelial cells (1). Within the hepatic sinusoids, lymphocyte adhesion is mediated through other adhesion proteins, such as ICAM-1 and VCAM-1, in addition to more specialized receptors, such as vascular adhesion protein-1 (VAP-1) and stabilin-1 (23, 33, 36).

Another family of proteins that may also play a role in modulating hepatic inflammation is the tetraspanin family. Tetraspanins comprise a large group of cell membrane proteins with low-sequence homology but conserved secondary and tertiary protein structures, with 33 distinct family members having been identified in mammals (11). They are characterized by four transmembrane domains delimiting three short intracellular regions and two extracytoplasmic loops, a small extracellular loop (EC1) and a large extracellular loop (EC2). CD151, also known as tetraspanin 24 (Tspan24), has previously been shown to cluster at the cell membrane in tetraspanin-enriched microdomains and is widely expressed in several cell types, including epithelial, endothelial, muscle, and hematopoietic cells (3, 20). In endothelial cells, CD151 was proposed to act as a lateral organizer and modulator of activities of transmembrane proteins, including ICAM-1 and VCAM-1, forming endothelial adhesive platforms (EAPs) (24). EAPs have been shown to play an essential role in the spatial organization of the membrane and in the recruitment of ICAM-1 and VCAM-1 toward the contact area with adherent leucocytes, thus regulating endothelial cell adhesiveness (5). Previous studies have demonstrated that CD151 contributes to the adhesion of leukocytes to human umbilical vein endothelial cells via EAPs (4). Our aim was to study whether CD151 contributed to lymphocyte recruitment to the human liver and whether it could be a novel target to treat chronic inflammatory liver disease and HCC.

In this study, we characterize the expression of CD151 in human liver tissue from a range of chronic liver diseases and in HCC. We show that CD151 is predominantly expressed on endothelial cells in the human liver. CD151 protein expression was found to be upregulated at sites of fibrosis in chronic liver diseases and overexpressed in HCC compared with normal liver tissue. We also demonstrate that HSECs and neovessels within the fibrotic septa account for the majority of CD151 expression observed in diseased livers in vivo. Isolated HSECs maintain strong expression of CD151, enabling studies into the regulation and function of CD151. We show that surface expression of CD151 in isolated HSECs can be upregulated by mimicking a tumorigenic environment and that CD151 colocalizes with VCAM-1 in stimulated HSECs. Finally, we confirm that CD151 mediates lymphocyte adhesion to primary HSECs in flow-based adhesion assays in a VCAM-1-dependent manner.

## MATERIALS AND METHODS

### 

#### Human tissue and blood samples.

All tissue and blood samples were obtained from the Queen Elizabeth Hospital, Birmingham, United Kingdom. Sections of normal human liver tissue were acquired from rejected donor liver tissue or from marginal tissue of hepatic resections. Diseased liver and HCC tissue was sourced from explanted livers during transplantation procedures. Tissue and blood samples from patients were obtained with written, informed consent and with local ethics committee approval (LREC reference 06/Q2702/61 South Birmingham, Birmingham, UK and 04/Q2708/41 South Birmingham, Birmingham, UK).

#### Immunohistochemistry.

Acetone-fixed, frozen liver sections (5 μm thick) were taken from normal liver, chronically diseased liver, and HCC tumor tissue, with at least four individual cases studied for each group. For immunohistochemical staining, endogenous peroxide activity was blocked with 0.3% H_2_O_2_ in methanol. The sections were then blocked for nonspecific antibody binding by using a 2× casein solution in PBS (from 10× stock; Vector) for 20 min. Slides were then incubated with primary antibody (mouse monoclonal anti-human CD151 IgG1, clone IIG5a; Abd Serotec; 5 μg/ml) or isotype-matched control (mouse IgG1-negative control, clone DAK-GO1; Dako; 5 μg/ml), diluted in PBS for 60 min at room temperature. The slides were then washed in PBS/0.1% Tween (PBST) and incubated with ImmPRESS Universal anti-mouse IgG reagent (Vector Laboratories) for 30 min at room temperature. Subsequently, slides were again washed with PBST and incubated with DAB Substrate (Vector) for 2 min. Sections were washed in tap water, counterstained with hematoxylin (Mayers; Pioneer Research Chemicals), dehydrated with sequential washes in alcohol and xylene, and finally mounted with DPX (phthalate-free) mounting medium (Cell Path). Images were acquired and analyzed on a Zeiss Axioscope microscope with concurrent Axiovision software (Carl Zeiss). Semiquantificative analysis of positively stained areas was performed with ImageJ software.

#### Immunofluorescence.

For immunofluorescent staining, acetone-fixed, frozen liver sections were initially blocked with 10% goat serum (GIBCO by Life Technologies) and 2× casein in PBS for 20 min at room temperature. Primary antibodies, or the relevant isotype-matched negative control, diluted in blocking buffer, were added and incubated for 60 min at room temperature. After being washed in PBST, the slides were incubated with the relevant fluorescence-conjugated goat anti-mouse secondary antibodies diluted in blocking buffer for 30 min. The sections were then washed again in PBST, and 300 nM DAPI (Sigma-Aldrich) was added to each section for 2 min before the slides were mounted in aqueous Fluorescent Mounting Medium (Dako) and imaged. Where colocalization staining was performed with two mouse IgG1 primary antibodies, CD151-targeting primary antibody and respective secondary antibody were added first, followed by extensive washing with PBST before sequential addition of the next mouse IgG1 primary and respective secondary antibody.

#### Antibodies.

Antibodies used, including concentration and source, are listed in Table 1.

**Table 1.** Monoclonal antibodies: clone, source, and final concentration

Monoclonal Antibodies	Clone	Source	Final Concentration, μg/ml
Mouse negative control	DAK-GO1	DAKO	6.7
Mouse negative control	DAK-GO5	DAKO	5.0
Mouse negative control	DAK-GO9	DAKO	5.0
Mouse anti-human CD151	IIG5a	Abd Serotec	6.7
Mouse monoclonal anti-actin α-smooth muscle	1A4	Sigma-Aldrich	5.0
Mouse monoclonal anti-human CD68	Y1/82A	BD Biosciences	5.0
Mouse monoclonal anti-human CD90	SE10	eBioscience	1.0
Mouse monoclonal anti-human CD31	JC70A	DAKO	5.0
Mouse monoclonal anti-human CD34	QBEND/10	Abd Serotec	5.0
Mouse monoclonal anti-human CD3	UCHT1	eBioscience	4.0
Mouse monoclonal anti-human EpCAM	HEA 125	Progen Biotechnik	5.0
Mouse monoclonal anti-human CK18	DC10	DAKO	1.0
Mouse anti-human early endosomes (EEA-1)	14	BD Biosciences	5.0
Mouse anti-human cis-Golgi (GM130)	35	BD Biosciences	5.0
Mouse anti-human lysosome (LAMP-1)	25	BD Biosciences	5.0
Mouse anti-human VCAM-1	BBA5	R&D Systems	5.0
Alexa Fluor 546 goat anti-mouse IgG2a	—	Life Technologies	8.0
Alexa Fluor 546 goat anti-mouse IgG2b	—	Life Technologies	8.0
Alexa Fluor 546 goat anti-rat IgG2a	—	Life Technologies	8.0

EpCAM, epithelial cell adhesion molecule.

#### Isolation of nonparenchymal cells.

A sample (30–50 g) of human liver tissue, taken from diseased livers (alcoholic liver disease, primary sclerosing cholangitis, primary biliary cholangitis) and normal liver resection margins, was mechanically chopped before being enzymatically digested with collagenase type 1A (10 mg/ml; Sigma-Aldrich). The resultant digest was filtered through a fine muslin mesh then centrifuged over a 33/77% (wt/vol) Percoll (GE Healthcare Life Sciences) density gradient for 20 min at 800 *g*. The nonparenchymal cell fraction was removed from the gradient interface, and cell populations were purified by immunomagnetic selection. Human epithelial antigen (HEA-125) antibody (50 μg/ml; Progen Biotechnik), along with conjugated with goat anti-mouse monoclonal antibody (mAb) Dynabeads (Invitrogen), was used to select biliary epithelial cells (BECs). HSECs were isolated by positive selection with anti-CD31 antibody-conjugated bead (Life Technologies) and cultured on rat-tail collagen-coated plastic ware. The remaining negatively selected activated liver myofibroblasts (aLMFs) were plated out into tissue culture plastic (16).

#### Isolation of human hepatic stellate cells.

Hepatic stellate cells (HSCs) were isolated from nonfibrotic liver tissue using pronase/collagenase digestion and buoyancy centrifugation, as previously described (16). Viability was tested by Trypan blue (Sigma-Aldrich) exclusion, and endogenous vitamin A autofluorescence was suggestive of a purity >90%.

#### Isolation of primary human hepatocytes.

Primary human hepatocytes (PHHs) were isolated using a modified two-stage collagenase procedure, as described previously (8).

#### Fluorescein isothiocyanate-labeled formaldehyde-denatured serum albumin uptake assay.

Fluorescein isothiocyanate-labeled formaldehyde-denatured serum albumin (FITC-FSA; Sigma-Aldrich) was diluted to 10 μg/ml in endothelial medium and added to HSEC monolayers grown in rat-tail collagen-coated 0.4 channel μ-Slides VI (Ibidi). After 15 min, the channels were washed three times in PBS, the cells were fixed with 4% paraformaldehyde solution for 10 min, washed with PBS, and DAPI stained to identify nuclei, and FITC positivity was analyzed using a Carl Zeiss LSM780 Zen Confocal microscope (Carl Zeiss) and LSM software. Control cells were incubated with the same medium without the addition of FITC-FSA. Purity was assessed as the percentage of FITC-positive plated cells taken from six random fields.

#### Cell lines and primary cell maintenance.

HepG2 cells were cultured in DMEM (GIBCO by Life Technologies) with 10% heat-inactivated FCS (ThermoFisher Scientific). BECs were cultured in Hams F12 Nutrient Mixture (GIBCO by Life Technologies) with the following supplements: 10% heat-inactivated human serum (TCS Biosciences), hepatocyte growth factor (HGF; 10 ng/ml; Peprotech), epidermal growth factor (EGF; 10 ng/ml; Peprotech), hydrocortisone (2 µg/ml; Sigma-Aldrich), cholera toxin (10 ng/ml; Sigma-Aldrich), tri-iodo-thyronine (2 nM; Sigma-Aldrich), insulin (0.124 U/ml; Sigma-Aldrich), 100 U/ml penicillin, 100 μg/ml streptomycin, 2 mM L-glutamine [penicillin-streptomycin-glutamine (100×); GIBCO by Life Technologies]. HSECs were maintained in human endothelial serum-free media (SFM) with 10% human serum, 100 U/ml penicillin, 100 μg/ml streptomycin, 2 mM L-glutamine (all GIBCO by Life Technologies), 10 ng/ml of HGF (Peprotech), and 10 ng/ml VEGF (Peprotech). HSCs and aLMFs were cultured in Dulbecco's Modified Eagle medium (DMEM; GIBCO by Life Technologies) with 16% heat-inactivated FCS (ThermoFisher Scientific) and 100 U/ml penicillin, 100 μg/ml streptomycin, and 2 mM L-glutamine (all GIBCO by Life Technologies).

#### Quantitative real-time PCR.

A sample (~20 mg) of liver tissue was homogenized in RLT buffer (QIAGEN) with β-mecaptoethanol (Sigma-Aldrich), on a gentleMACS Dissociator (Miltenyi Biotec).

RNA extraction was then performed using the RNeasy Mini Kit (QIAGEN) following the manufacturer’s protocol with column DNaseI digestion. Alternatively, RNA was extracted from highly pure cell populations isolated from a range of livers, including alcoholic liver disease, primary sclerosing cholangitis, primary biliary cirrhosis, and nonalcoholic steatohepatitis. Additionally, HSECs were grown to confluence in six-well plates (Corning CoStar), stimulated for 24 h (see *Stimulation of HSECs* below), then harvested by direct cell lysis through the addition of RLT buffer. RNA extraction was performed with the use of an RNeasy Micro Kit (QIAGEN), following the manufacturer’s protocol as above.

Following RNA extraction, RNA concentration and purity were determined by use of a Nanophotometer (Implen), and cDNA was generated using SuperScriptIII Reverse Transcriptase (Invitrogen). To validate our hepatocyte results, a commercially available cDNA generated from human hepatocytes was also purchased (Caltag Medsystems) and analyzed for CD151 expression. Assessment of mRNA expression levels was performed via quantitative real-time PCR (qRT-PCR), using predesigned TaqMan Gene Expression Assays (Applied Biosystems) and 2× TaqMan Universal PCR Master Mix (Applied Biosystems). qRT-PCR was performed on a Lightcycler 480 (Roche) using the following cycling conditions: 95°C for 10 min followed by 45 cycles of 95°C for 10 s, 60°C for 1 min, 72°C for 1 s. Samples were run in triplicate with average cycle threshold (C*t*) values for CD151 normalized against an appropriate housekeeping gene. Different housekeeping genes were used in separate experiments to ensure a consistent expression across the disease states or cell treatments being studied. Primers used are listed in Table 2.

**Table 2.** Target and TaqMan assay identification

Target	TaqMan Assay ID
*CD151*	Hs00911635_g1
*GAPDH*	Hs99999905_m1
*18S*	Hs99999901_s1
*GUSB*	Hs00939627_m1
β-Actin	Hs01060665_g1

#### Western blot analysis.

A sample (~75 mg) of liver tissue was homogenized in 1.5 ml CelLytic MT lysis buffer (Sigma-Aldrich) with 1% protease inhibitor cocktail (Sigma-Aldrich), 1% phosphatase inhibitor cocktail 3 (Sigma-Aldrich), and 5 U/ml DNase-I (Sigma-Aldrich), using gentleMACS M-tubes (Miltenyi Biotec). Lysate protein concentrations were calculated and normalized using the bicinchoninic acid assay (Sigma-Aldrich) per standard protocols, with BSA (Sigma-Aldrich) utilized as a protein standard. Twenty micrograms of protein lysate was resolved via SDS-PAGE on a 12% polyacrylamide gel and subsequently transferred to a nitrocellulose membrane (ThermoFisher Scientific) by semi-dry transfer using the Trans-Blot Turbo Transfer System (Bio-Rad). Following transfer, membranes were blocked in 5% nonfat milk (Marvel) in TBS/0.1% Tween (TBST) for 1 h at room temperature. Primary antibodies (CD151; Abd Serotec; 1.4 μg/ml and β-actin; Sigma-Aldrich; 1 in 1,000 dilution), diluted in TBST with 3% BSA (GIBCO by Life Technologies) and 0.1% azide (Sigma-Aldrich) were then added to the membranes and incubated overnight at 4°C. Secondary antibodies [goat anti-mouse horseradish peroxidase (HRP); Dako; 0.14 μg/ml and goat anti-mouse IRDye 680LT; LI-COR; 0.14 μg/ml] were diluted in 5% nonfat milk and added for 60 min at room temperature. Finally the membranes were developed with enhanced chemiluminescent substrate (ECL; Pierce) and Amersham Hyperfilm MP (GE Healthcare) or, where IRDye secondary antibodies were used, scanned on an Odyssey Imaging System (Li-COR Biosciences). Semiquantitative analysis of protein bands was performed on ImageJ software, with CD151 expression shown relative to the housekeeping protein, β-actin.

#### Stimulation of HSECs.

HSECs were stimulated with a range of cytokines and growth factors. In each case, cells were incubated in SFM with 10% FCS for 24 h before respective stimuli were added for the subsequent 24 h. Where HepG2 supernatant was used to stimulate HSECs, HepG2 cells were grown to confluence in DMEM with 10% FCS before the media was exchanged for SFM with 10% FCS for 24 h. The resultant supernatant was then used to stimulate primary HSEC cultures for 24 h. Control isolates consisted of HSECs maintained in human endothelial SFM with 10% FCS for 24 h. Growth factors (VEGF and HGF) and proinflammatory cytokines (TNF-α and IFN-γ) used in HSEC stimulation experiments were used at a concentration of 10 ng/ml and purchased from Peprotech.

#### HSECs in hypoxia.

Where the effect of hypoxic conditions on CD151 expression in HSECs was investigated, HSEC media was degassed for 24 h in a Whitley H35 hypoxystation hypoxic chamber (Don Whitley Scientific) at 1% O_2_ before HSECs were incubated in the chamber with the degassed media for 24 h. Cells were then fixed in cold methanol in the chamber and subsequently analyzed via ELISA (see *Cell-based ELISA* below).

#### Cell-based ELISA.

HSECs were grown to confluence in rat tail collagen-coated 96-well plates (Corning CoStar) and then stimulated (see *Stimulation of HSECs*), before being fixed in 100% methanol and washed with a buffer solution of PBS with 0.1% BSA (Sigma-Aldrich). Cells were then blocked for nonspecific antibody binding in PBS with 2% goat serum diluted for 45 min. Primary antibody (CD151; Abd Serotec; 5 μg/ml) or mouse IgG1 negative control (clone DAK-GO1 DAKO; 5 μg/ml) were added to the appropriate wells and incubated for 60 min at room temperature. This was followed by washing with PBS/BSA solution and then the addition of goat anti-mouse HRP-conjugated secondary antibody (Dako; 0.2 μg/ml) to each well for 45 min. The cells were then washed again with PBS/BSA before developing with an OPD substrate (1,2-phenylenediamine dihydrochloride tablets; Dako) dissolved in distilled water with 2.5 μl of 30% H_2_O_2_. The reaction was arrested with a stop solution of 0.5 M H_2_SO_4_ (Sigma-Aldrich) before absorbance was measured on a Synergy HT automated microplate reader (BioTek) at 490 nm. Mean absorbance values were calculated from triplicate wells and corrected for background absorbance by subtracting absorbance values obtained from triplicate wells of an isotype-matched negative control antibody.

#### Immunocytochemistry.

For immunofluorescent staining of HSECs, cells were grown to confluence on rat tail collagen-coated eight-well glass-bottom μ-slides (Ibidi), stimulated (see *Stimulation of HSECs*) and subsequently fixed in 100% methanol. The cells were then blocked and permeabilized with a solution of PBS with 10% goat serum, 2× casein, and 0.1% Triton-X 100 (Sigma-Aldrich) for 20 min at room temperature. Primary antibodies, diluted in blocking buffer, were then added and incubated for 60 min, followed by isotype-specific fluorescence-conjugated goat anti-mouse secondary antibodies for 30 min. Finally, 300 nM DAPI was added to each chamber for 2 min before the mounting in aqueous Fluorescent Mounting Medium (Dako). All immunofluorescent staining was imaged on a Carl Zeiss LSM780 Zen Confocal microscope (Carl Zeiss) and analyzed by LSM software.

#### Peripheral blood lymphocyte isolation.

Blood samples were taken from patients undergoing venesection for hemochromatosis, which we have previously used for lymphocyte migration assays (33). Lymphocytes were isolated by density-gradient centrifugation over Lympholyte (Cedarlane Laboratories) for 25 min at 800 *g*, before platelet and monocyte depletion. These steps ensure highly pure populations of peripheral blood lymphocytes (PBLs) as previously described (22). These cells were washed and resuspended in RPMI 1640 containing 10% FCS.

#### Static adhesion assays.

Tissue sections from explant livers from alcoholic liver disease, primary sclerosing cholangitis, primary biliary cholangitis, and samples of normal liver from resections were initially incubated with blocking antibody to CD151 (IIG5a; AbD Serotec; 10 μg/ml) or control antibody. The sections were then overlaid with PBLs that had been prelabelled with cell tracker green (ThermoFisher Scientific); PBLs were resuspended at 1 × 10^6^/ml, and 100-μl aliquots were added to each section for 30 min at room temperature to adhere in static conditions. The nonadherent cells were removed, and the remaining adherent cells were fixed with acetone. The sections were then stained with DAPI and imaged on a Carl Zeiss LSM780 Zen Confocal microscope (Carl Zeiss), and lymphocytes present in a minimum of 10 representative high-power fields per section were counted.

#### Flow-based adhesion assays.

In vitro flow-based adhesion assays previously described by our laboratory were used to investigate the role of CD151 on HSECs under conditions of physiological flow (32). Briefly, HSECs were seeded in channels of rat tail collagen-coated 0.4 Channel μ-Slides VI (Ibidi) and cultured to confluence overnight. Cells were then stimulated with TNF-α for 24 h to promote adhesion receptor expression. A sample (1 × 10^6^ cells/ml) of PBLs, in flow media comprising RPMI with 0.1% BSA, was then perfused over the HSECs at a physiological shear stress of 0.05 Pa. Each chamber of the microslides was perfused for 5 min with PBLs before 5 min of wash with the flow media alone. The subsequent adherent and migratory cells were then imaged by phase-contrast microscopy with an Olympus IX50 Inverted Microscope (Olympus) connected to an image capture device, with video recordings of 12 frames from each chamber taken. Blinded analysis was then performed for quantification of the number of adherent or transmigrated PBLs. The number of adherent cells were normalized to cells per square millimeter per 10^6^ cells perfused, and the number of transmigrated cells was expressed as a percentage of total adherent cells. The addition of blocking antibodies to CD151 (IIG5a; AbD Serotec; 10 μg/ml), VCAM-1 (BBA5; R&D Systems; 10 μg/ml), or isotype-matched negative control antibodies were performed immediately preceding each assay. Each antibody was diluted in flow media before 50 μl was added to each chamber and incubated for 30 min before initiation of the flow assay.

#### siRNA knockdown of CD151 in HSECs.

Cells were transiently transfected with 25 nM HsCD151_5 Flexitube siRNA (S102777250; QIAGEN) or a nontargeting siRNA control (negative control siRNA; QIAGEN), using Lipofectamine RNAiMAX Transfection Reagent (Invitrogen). Briefly, 2.5 × 10^5^ or 7.5 × 10^4^ HSECs were seeded in rat tail collagen-coated six-well culture plates or 0.4 Channel μ-Slides VI (Ibidi), respectively, and grown to confluence overnight. Subsequently, siRNA duplexes diluted in Opti-MEM (GIBCO by Life Technologies) were mixed with a final concentration of 0.3% Lipofectamine RNAiMAX and incubated for 10 min at room temperature. Cells were then washed twice with PBS, and the duplex/Lipofectamine RNAiMAX mixture was added to the cells and incubated for 4 h at 37°C. The duplex/Lipofectamine RNAiMAX mixture was then removed from the cells and replaced with HSEC medium without antibiotics and cultured as normal. After 24 h, transfected cells were stimulated with 10 ng/ml TNF-α and left for a further 24 h. HSECs cultured in six-well culture plates were harvested in CelLytic MT lysis buffer with 1% protease inhibitor, 1% phosphatase inhibitor cocktail 3, and 5 U/ml DNase-I, and CD151 knockdown was confirmed via Western blot analysis (see *Western blot analysis* above). HSEC-cultured 0.4 Channel μ-Slides VI were used in flow-based adhesion assays (see *Flow-based adhesion assays* above).

#### Statistical analysis.

All results are presented as means ± SE or median value ± interquartile range (IQR) where the data form a nonparametric distribution. Statistics were performed on Prism 6.0 software (GraphPad), with Student’s *t*-tests (parametric) and Mann-Whitney *U*-tests (nonparametric) used to compare statistical significance between two groups. One-way ANOVA with post hoc Tukey’s (parametric) test and Kruskall-Wallis one-way ANOVA with post hoc Dunn’s test (nonparametric) were also used when comparison between multiple groups was required. Values of *P* < 0.05 were considered statistically significant.

## RESULTS

### 

#### CD151 is upregulated in chronic liver disease and hepatocellular cancer.

**Fig. 1.**
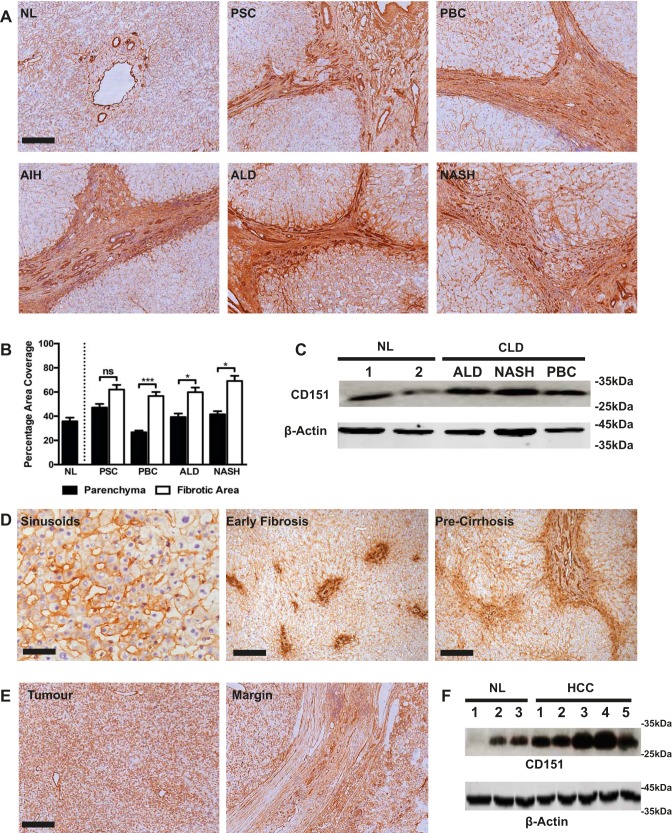
CD151 expression is upregulated in chronic diseased livers and hepatocellular cancer. *A*: immunohistochemical staining of CD151 (brown) in representative examples of normal liver (NL), primary sclerosing cholangitis (PSC), primary biliary cholangitis (PBC), autoimmune hepatitis (AIH), alcoholic liver disease (ALD), and nonalcoholic steatohepatitis (NASH). *B*: surface area quantification of immunohistochemical staining in parenchymal and fibrotic regions; *n* = 4 in each group. *C*: Western blot analysis of CD151 in normal liver and chronic liver disease (CLD) samples. *D*: immunohistochemical staining (brown) of CD151 in liver sinusoids and in precirrhotic liver sections in patients with PSC. *E*: immunohistochemical staining (brown) of CD151 in tumor core and tumor margin in a representative case of hepatocellular carcinoma (HCC). *F*: Western blot analysis of CD151 in normal liver and HCC samples. *P < 0.05, ***P < 0.005; bar 50 = μm (*D*, *left*), 200 μm (*A*; *D*, *middle* and *right*; and *E*).

Immunohistochemical analysis of normal and diseased human liver tissue revealed that CD151 localized to the hepatic sinusoids and major vasculature with only weak expression on bile ducts and hepatocytes (Fig. 1*A*). Although expression of CD151 in the liver parenchyma was similar in normal and diseased states, more CD151 was detected in fibrotic septa (Fig. 1, *A* and *B*), with particularly strong expression on scar-associated vessels. Western blotting confirmed upregulation of CD151 at the protein level in diseased tissue. (Fig. 1*C*). Additional staining of liver sections from patients with earlier stages of fibrosis demonstrated strong expression of CD151 on the sinusoids, and we also noted upregulation of CD151 in areas of fibrosis (Fig. 1*D*).

Primary human HCC tumor tissue stained positive for CD151 throughout the tumor sinusoids, on the tumor-associated vasculature, and on the tumor cells themselves (Fig. 1*E*). Also, at the tumor margin, CD151 expression was evident within the tumor capsule and on capsule-associated vessels (Fig. 1*E*). Increased tumoral expression of CD151 was confirmed by Western blot analysis (Fig. 1*F*).

#### CD151 is expressed predominantly in HSECs and neovessels in chronic liver disease.

**Fig. 2.**
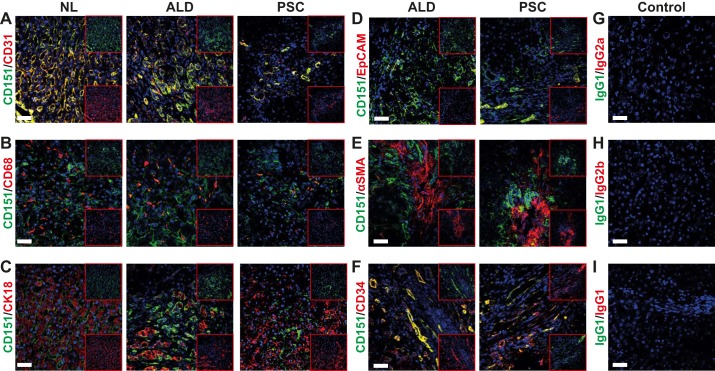
CD151 colocalizes to endothelial markers within the hepatic sinusoids and on neo vessels in the fibrotic septum. *A*–*C*: dual-color immunofluorescent staining of liver tissue samples from normal liver and chronically inflamed livers (ALD and PSC) for CD151 (green) with endothelial marker CD31 (*A*), macrophage marker CD68 (*B*), hepatocyte marker CK18 (C). *D*–*F*: dual-color immunofluorescent staining of liver tissue samples of chronically inflamed livers (ALD and PSC) for CD151 (green) with biliary epithelial cell adhesion molecule (EpCAM) (*D*), fibroblast marker α-smooth muscle actin (SMA) (*E*), and neovessel marker CD34 (*F*). *G*–*I*: dual-color immunofluorescent staining of liver tissue sample isotype-matched controls. Bar = 50 μm.

Dual-color immunofluorescent colocalization was used to determine cell-specific expression of CD151 in normal and chronically diseased (ALD and PSC) liver tissues. CD151 was found to colocalize with the endothelial marker CD31 within the sinusoids (Fig. 2*A*) but not CD68-positive Kupffer cells (Fig. 2*B*). CD151 expression also demonstrated weak colocalization with the hepatocyte marker CK18 and biliary epithelial cell adhesion molecule (Fig. 2, *C* and *D*). Furthermore, CD151 was not expressed by activated stellate cells (α-smooth muscle actin-positive; Fig. 2*E*) but was associated with CD34-positive cells in tissue taken from chronically inflamed livers (Fig. 2*F*), indicating expression by vascular endothelium and neovessels at sites of inflammation and fibrotic septa.

**Fig. 3.**
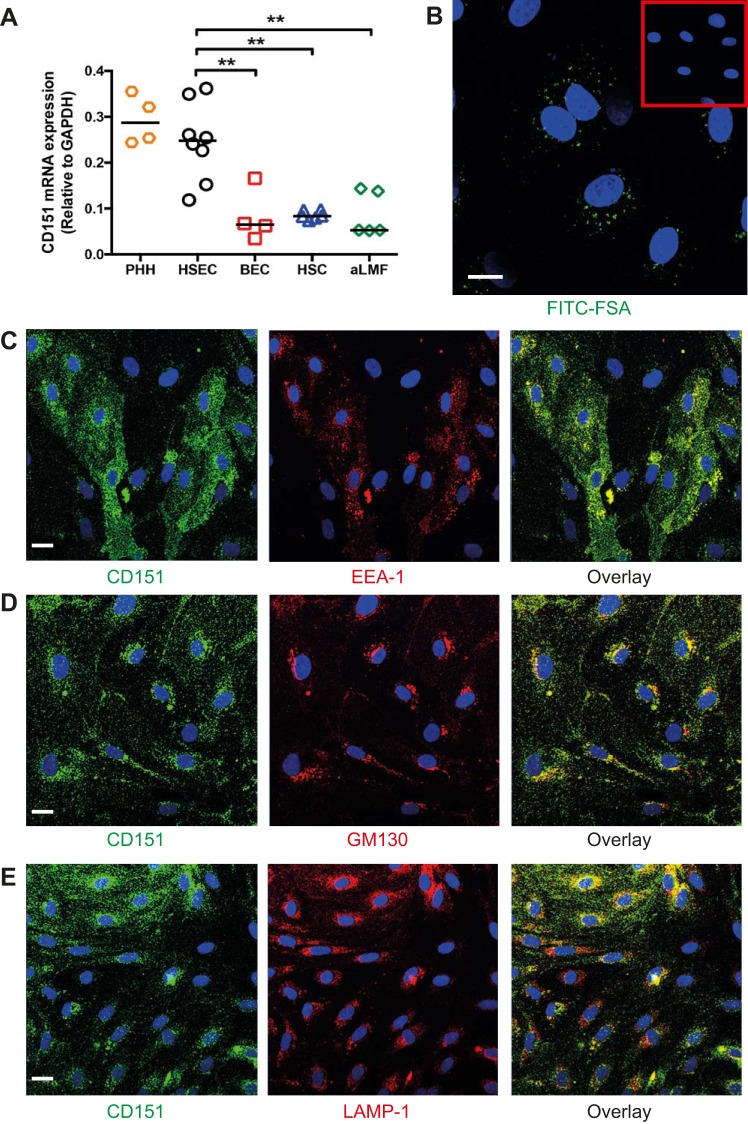
CD151 is highly expressed in isolated primary human liver sinusoidal endothelial cells. *A*: mRNA levels of CD151 in populations of isolated primary human hepatocytes (PHH), human hepatic sinusoidal endothelial cells (HSEC), biliary epithelial cells (BEC), hepatic stellate cells (HSC), and activated liver myofibroblasts (aLMF). *B*: confocal microscopy of HSECs after incubation with FITC-FSA for 15 min. *Inset*: control cells. *C*–*E*: dual-color immunofluorescent staining of HSECs for CD151 (green) and early endosome marker (EEA-1) (*C*), Golgi marker GM130 (*D*), and lysosome marker LAMP-1 (*E*). Bar = 20 μm (*B*), 15 μm (*C*–*E*). **P < 0.01.

We next isolated primary human liver cell populations from fresh tissue, including PHHs, HSECs, BECs, HSCs, and aLMFs. We found that CD151 expression at the transcript level was similar between HSECs and hepatocytes but significantly higher compared with the other cell types (Fig. 3*A*). We have previously shown that sinusoidal endothelial cells isolated from human liver tissue maintain their distinct phenotype typified by high scavenger receptor expression and atypical junctions compared with conventional endothelium (29). These cells were isolated from various diseases and stages of liver disease; however, detailed analysis shows that in culture each isolate is phenotypically very similar with comparable expression levels of adhesion receptors and junctional molecules (29). Confirmation of the phenotype and purity of HSECs were determined by rapid uptake of FITC-FSA. Our isolation method routinely provided a sinusoidal endothelial purity of >85% of cells, with all cells expressing CD31 as determined by flow cytometry (Fig. 3*B* and materials and methods). Immunofluorescent staining of CD151 in HSECs showed that CD151 colocalized with early endosomes, Golgi apparatus, and lysosomes (EEA-1, GM130, and LAMP-1, respectively) (Fig. 3, *C*–*E*). These results suggest that CD151 undergoes continual cell cycling in HSECs, with its posttranslational modification in the Golgi apparatus before transportation to the cell surface via endosomes and subsequent degradation within lysosomes.

#### Regulation of CD151 in HSECs.

**Fig. 4.**
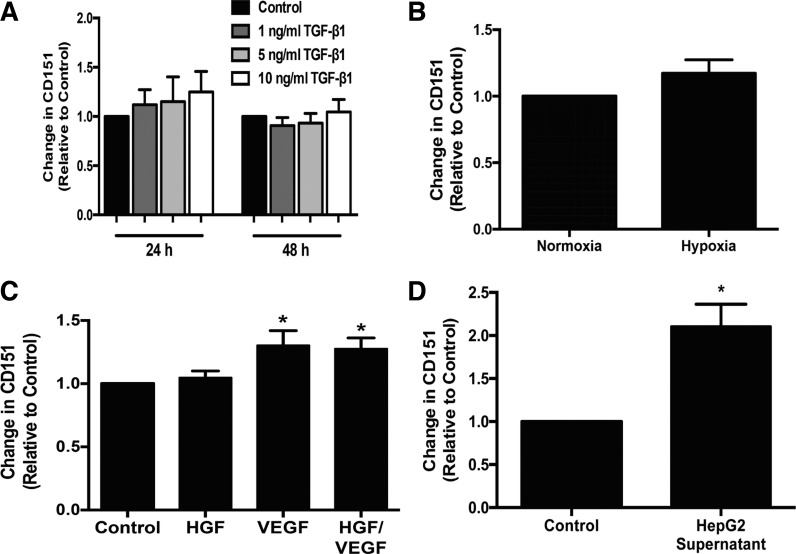
CD151 is upregulated by growth factors and supernatant from hepatocellular cancer cell line. Expression of CD151 by ELISA in HSECs treated with transforming growth factor-β (TGF-β) (*A*). Each sample was run in triplicate; *n* = 3. *B*: expression of CD151 by ELISA in HSECs cultured in normoxic and hypoxic conditions; *n* = 6. *C*: expression of CD151 by ELISA in HSECs treated with hepatocyte growth factor (HGF) and VEGF with each sample run in triplicate; *n* = 6. *D*: expression of CD151 by ELISA in HSECs treated with HepG2 supernatant with each sample run in triplicate; *n* = 4. *P < 0.05.

Having detected increased endothelial expression of CD151 at sites of chronic liver injury, we assessed the regulation of the protein on HSECs by proinflammatory cytokines (TNF-α and IFN-γ) and the fibrogenic cytokine, IL-13. Stimulation with these cytokines did not alter the expression of CD151 in HSECs (data not shown). Chronic fibrosis is also associated with increased transforming growth factor β (TGF-β) activity (30) and tissue hypoxia, which contributes to angiogenesis (28), leading us to study the effect of prolonged TGF-β stimulation as well as culture under hypoxic conditions on expression of CD151 by HSECs. We did not detect any increase in CD151 protein expression with TGF-β stimulation or hypoxia (Fig. 4, *A* and *B*). Previously, our group has shown that growth factors can increase the expression of atypical adhesion receptors, such as CLEVER-1/stabilin-1 (33). Stimulation with growth factors involved in angiogenesis, HGF and VEGF, led to a statistically significant increase in expression of CD151 protein with VEGF stimulation; there was no additional increase in expression with the combination of growth factors (Fig. 4*C*). We detected endothelial CD151 at high levels on HCC tumor vessels and in vessels within the stroma surrounding the tumor (Fig. 1*C*), leading us to hypothesize that factors produced locally by the tumor may promote CD151 expression. To study this, HSECs were incubated with supernatant from an HCC cell line (HepG2), which led to significant upregulation of CD151 expression in HSECs compared with basal conditions, which was greater than that observed for treatment with HGF and VEGF (Fig. 4*D*).

#### CD151 is a regulator of VCAM-1-mediated adhesion of lymphocytes to HSECs.

**Fig. 5.**
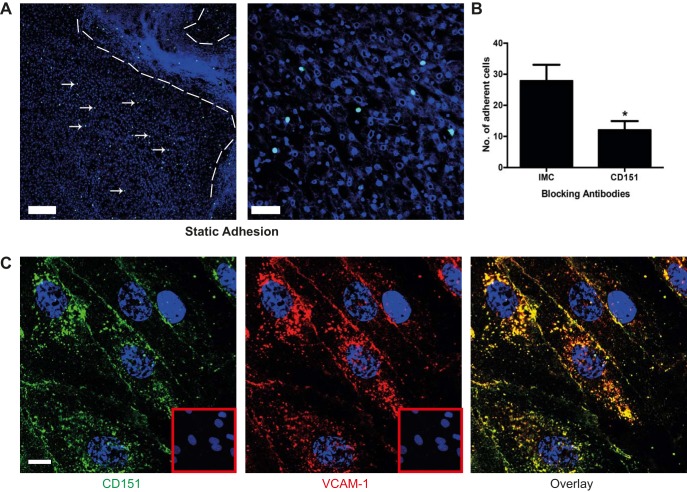
CD151 mediates lymphocyte binding to human liver tissue and colocalizes with VCAM-1 in primary human liver endothelium. *A*: representative low- and high-power images of static Stamper–Woodruff adhesion assays of cell tracker green-labeled peripheral blood lymphocytes binding to human liver tissue demonstrating adhesion to the sinusoidal regions within the liver parenchyma; cell nuclei were stained with DAPI. Dashed lines outline a fibrous septum, and arrows highlight lymphocytes within the parenchyma. *B*: sections were treated with anti-CD151 antibody or appropriate class-matched control antibody, and the results of 4 independent experiments are shown as the number of adherent cells per low-power image. *C*: dual-color immunofluorescent staining of TNF-α-treated HSECs with CD151 (green), VCAM-1 (red), and DAPI (nucleus, blue). *Insets*: background fluorescence for each class-matched control antibody. Bar = 200 μm (*A*), 50 μm (*B*), 10 μm (*C*). *P < 0.05.

Modified Stamper-Woodruff tissue binding assays on diseased liver tissue demonstrated that lymphocytes bound to the fibrotic septa where we had shown CD151 was upregulated, as well as within the sinusoidal channels throughout the parenchyma (Fig. 5*A*). Antibody blockade of CD151 significantly inhibited lymphocyte adhesion to human liver tissue compared with an isotype-matched control (Fig. 5*B*). Lymphocyte recruitment in the hepatic sinusoids is selectin independent, and a major role in lymphocyte capture and adhesion to HSECs is played by VCAM-1 (23, 37). Subsequent transmigration is a multistep process involving ICAM-1, VAP-1, and stabilin-1 (23, 33, 37). Previous studies have reported that CD151 is associated with VCAM-1 and ICAM-1 (5), leading us to look for colocalization of CD151 with VCAM-1 in HSECs. We were able to show CD151 colocalization with VCAM-1 in stimulated HSECs both at the cell membrane and within intracellular compartments (Fig. 5*C*).

**Fig. 6.**
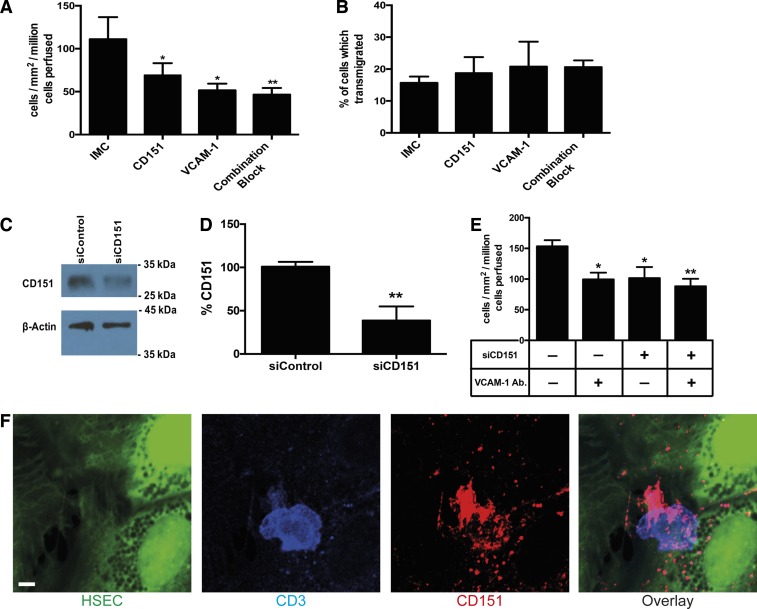
CD151 mediates the adhesion of peripheral lymphocytes to HSECs under conditions of shear stress. *A*: quantification of adherence of lymphocytes to monolayers of HSECs in flow assays pretreated with isotype-matched control (IMC) and CD151, VCAM-1, and a combination of CD151/VCAM-1-blocking antibodies; *n* = 3 independent experiments with different HSECs. *B*: proportion of adherent cells that underwent transendothelial migration after blockade with IMC, CD151, VCAM-1, and a combination of CD151/VCAM-1; *n* = 3 independent experiments with different HSECs. *C*: representative Western blot analysis of CD151 in HSECs treated with control and CD151 siRNA knockdown. *D*: quantification of CD151 expression in HSECs treated with siRNA knockdown of CD151 expressed as a % of expression in control HSECs; *n* = 4 independent experiments with different HSECs. *E*: quantification of adherence of lymphocytes to monolayers of HSECs in flow assays pretreated with control siRNA and CD151 siRNA, VCAM-1, and a combination of CD151/VCAM-1-blocking antibodies; *n* = 4 independent experiments with different HSECs. *F*: representative confocal image of CD151 (red) accumulation around a CD3-positive lymphocyte (blue) firmly adherent to cell tracker green-labeled HSECs. Bar = 5 μm, *P < 0.05, **P < 0.01.

To understand the role of CD151 in the adhesion cascade during lymphocyte recruitment to the liver, we performed lymphocyte-endothelial adhesion assays under physiologically relevant flow rates and determined the effect of blocking CD151 and VCAM-1 both independently and in combination. Blockade of CD151 alone led to a significant reduction in total lymphocyte adhesion to HSECs (Fig. 6*A*), reducing adhesion to levels similar to those observed for blockade of the typical adhesion molecule, VCAM-1. However, blocking both receptors did not reduce adhesion, further suggesting that CD151 cooperates with VCAM-1 to regulate lymphocyte adhesion to HSECs (Fig. 6*A*). We used phase-contrast microscopy during the flow adhesion assay to determine the proportion of lymphocytes adherent to the surface of the endothelial cells and those that had migrated underneath the endothelial monolayer (32). CD151 blockade did not inhibit the proportion of cells migrating underneath the endothelial monolayer, suggesting that it does not contribute to the transendothelial migration step in lymphocyte recruitment across HSECs (Fig. 6*B*). As an alternative to antibody blockade, we also inhibited CD151 function by knocking down expression with siRNA (Fig. 6*C*). We achieved ~60% knockdown of CD151 (Fig. 6*D*), and repeat flow assays confirmed a functional role for CD151 in lymphocyte adhesion to HSECs (Fig. 6*E*). Once again, CD151 inhibition and VCAM-1 blockade had similar effects on reducing adhesion, with no additional inhibition when CD151 knockdown and VCAM-1 blockade were combined (Fig. 6*E*). Using a fixed cell technique with confocal microscopy and immunofluorescent staining that we recently described (33), we visualized cellular CD151 on HSECs in relation to lymphocyte binding from flow. CD151 enrichment was seen around adherent CD3+ lymphocytes, consistent with a role of CD151 in immune cell recruitment (Fig. 6*F*).

## DISCUSSION

Tetraspanins are a family of proteins that contribute to several aspects of cell-cell interactions. CD151 is involved in cell-to-cell communication, wound healing, platelet aggregation, cell trafficking, and angiogenesis (7, 15, 34, 38). In addition, it has been reported to play a role in leukocyte adhesion to human umbilical vein endothelial cells. To our knowledge, a role for CD151 in leukocyte recruitment to specialized vascular beds has not been investigated. A key step in leukocyte recruitment to the liver is binding to HSECs. These endothelial cells have a distinct phenotype compared with conventional venular endothelium determined by the functional requirements of their microenvironment (9, 14). We have previously shown that, following isolation from human liver tissue, these endothelial cells maintain their unique phenotype and junctional complexes as well as their functional characteristics (29). In the present study, we have characterized the expression of CD151 in normal and diseased human liver and found it to be upregulated at sites of leukocyte recruitment, including the hepatic sinusoids and neovessels of the fibrous septa as well as on vessels in HCC and surrounding peritumoral stroma. Isolated HSECs maintain expression of CD151, allowing us to study the regulation and function of CD151 in these cells. Detailed analysis of CD151 cellular distribution suggests that it is mobilized to the cell surface from intracellular vesicles, and, although we detected the protein at chronic inflammatory sites in vivo, expression of CD151 was not regulated in vitro by the proinflammatory cytokines TNF-α or IFN-γ in contrast to many of the standard endothelial adhesion receptors. Previous studies on epithelial cells have demonstrated that CD151 is upregulated by chronic exposure to TGF-β (hepatocyte cell line) (17) and suppressed by hypoxia (colon cancer cell line) (12). In our study, neither stimulation of HSECs with TGF-β nor exposure to hypoxia altered CD151 expression, suggesting that the regulation of CD151 differs between endothelial cells and epithelial cells. However, expression was increased by treatment with growth factors, specifically VEGF, and by culture in culture supernatant from an HCC cell line. These findings suggest that CD151 in endothelial cells is regulated by microenvironmental signals induced by tissue remodeling or those present in the tumor microenvironment.

We then demonstrated that CD151 mediates lymphocyte adhesion to human liver tissue and to HSECs under flow. CD151 was closely associated with VCAM-1 on HSECs, and the fact that functional blockade of VCAM-1 and CD151 in combination was not additive suggests that CD151 mediates lymphocyte adhesion to HSECs through its interaction with VCAM-1. Stereotactical interference of CD151 blocking antibody on lymphocyte binding to VCAM-1 was ruled out by demonstrating that CD151 knockdown by siRNA also significantly inhibited lymphocyte binding to HSECs.

Together, these results suggest that CD151 may play a particular role in maintaining lymphocyte recruitment in chronic liver injury, leading to persistent inflammation and ongoing tissue damage and fibrogenesis. This makes CD151 a possible therapeutic target for inflammatory liver disease. Despite the growing global burden of chronic inflammatory liver disease, treatment strategies mainly rely upon removal of the underlying causative agent or transplantation for end-stage disease. By interfering with the ability of CD151 to maintain lymphocyte recruitment in chronic liver disease, it may be possible to switch off persistent inflammation, leading to resolution. Tetraspanin interactions can affect heterotypic partner protein receptor avidity, as shown by Ke et al. (18), who used an antibody to dissociate CD151 from an integrin partner protein, which prevented tumor progression. VCAM-1 is a receptor that is not restricted to endothelial cells but also found on stromal cells and epithelial cells, where it can regulate lymphocyte adhesion and survival (2, 27, 35). Thus modulating CD151 interactions with VCAM-1 in endothelial cells may be an attractive and specific target for chronic inflammatory liver disease. Our observations that CD151 is broadly upregulated in HCC and that factors secreted by HCC can induce expression on endothelium suggest that it may have an important role in hepatic tumorigenesis. What role CD151 plays is presently unclear. It could promote tumorigenesis as a consequence of establishing persistent chronic inflammation, or, as has been reported in other systems, it may promote the growth and spread of tumor cells. Finally, by modulating lymphocyte recruitment, it may alter the immune response to the tumor. It is also now well accepted that the immune system and immune cell recruitment make a major contribution to the development and prognosis of HCC, with immunotherapy already commenced in clinical trials (25). Thus we propose that CD151 could be targeted to inhibit lymphocyte recruitment to the liver and prevent the progression of inflammatory liver diseases to end-stage fibrosis and reduce the risk of HCC.

## GRANTS

S. Shetty was funded by a Wellcome Trust Intermediate Clinical Fellowship (097162/Z/11/Z). D. Patten and S. Shetty were also funded by the Queen Elizabeth Hospital Birmingham Charity. C. Weston and E. Shepherd were funded by a Wellcome Trust Programme grant (091019/Z/09/Z). J. Wadkin and S. Kamarajah were funded by bursaries from Cancer Research UK. This paper presents independent research supported by the NIHR Birmingham Liver Biomedical Research Unit based at the University Hospitals Birmingham NHS Foundation Trust and the University of Birmingham. The views expressed are those of the authors and not necessarily those of the NHS, the NIHR, or the Department of Health.

## DISCLOSURES

No conflicts of interest, financial or otherwise, are declared by the authors.

## AUTHOR CONTRIBUTIONS

D.A.P., F.B., C.J.W., and S.S. conceived and designed research; J.C.W., D.A.P., S.K., E.L.S., and V.N. performed experiments; J.C.W., D.A.P., S.K., E.L.S., and C.J.W. analyzed data; D.A.P. and C.J.W. interpreted results of experiments; J.C.W., D.A.P., D.H.A., C.J.W., and S.S. drafted manuscript; D.A.P. and S.S. prepared figures; D.A.P. edited and revised manuscript; F.B., D.H.A., and C.J.W. approved final version of manuscript.
